# Incidence of leukemias in children from El Salvador and Mexico City between 1996 and 2000: Population-based data

**DOI:** 10.1186/1471-2407-5-33

**Published:** 2005-04-04

**Authors:** Juan Manuel Mejía-Aranguré, Miguel Bonilla, Rodolpho Lorenzana, Servando Juárez-Ocaña, Gladys de Reyes, María Luisa Pérez-Saldivar, Guadalupe González-Miranda, Roberto Bernáldez-Ríos, Antonio Ortiz-Fernández, Manuel Ortega-Alvarez, María del Carmen Martínez-García, Arturo Fajardo-Gutiérrez

**Affiliations:** 1 Clinical Epidemiology, Pediatric Hospital, Centro Médico Nacional "Siglo XXI", Mexico City, Mexico; 2 Hematology-Oncology, Hospital Nacional de Niños "Benjamín Bloom", San Salvador, El Salvador; 3 Department of Pediatrics, School of Medicine, Universidad de El Salvador, San Salvador, El Salvador; 4 Laboratory of Immunopathology, Guatemala City, Guatemala; 5 Hematology, Pediatric Hospital, Centro Médico Nacional "Siglo XXI", Mexico City, Mexico; 6 Hematology, General Hospital, Centro Médico Nacional "La Raza", Mexico City, Mexico

## Abstract

**Background:**

There are very few studies that report the incidence of acute leukemias in children in Latin America. This work assesses the incidence of acute leukemias, between 1996 and 2000, in children from 0–14 years old who were attended at the Mexican Social Security Institute in Mexico City and in children from 0–11 years old in El Salvador.

**Methods:**

Design: Population-based data. Hospitals: In San Salvador, El Salvador, Hospital Nacional de Niños "Benjamín Bloom", the only center in El Salvador which attends all children, younger than 12 years, with oncologic disease. The Pediatric Hospital and the General Hospital of the Mexican Social Security Institute in Mexico City, the only centers in Mexico City which attend all those children with acute leukemia who have a right to this service. Diagnosis: All patients were diagnosed by bone marrow smear and were divided into acute lymphoid leukemia (ALL), acute myeloid leukemia (AML), chronic myeloid leukemia (CML), and unspecified leukemias (UL). The annual incidence rate (AIR) and average annual incidence rate (AAIR) were calculated per million children. Cases were stratified by age and assigned to one of four age strata: 1) <1 year; 2) 1–4 years; 3) 5–9 years, or 4) 10–14 or 10–11 years, for Mexico City and El Salvador, respectively.

**Results:**

The number of cases was 375 and 238 in El Salvador and Mexico City, respectively. AAIRs in Mexico City were 44.9, 10.6, 2.5, 0.5, and 58.4 per million children for ALL, AML, CML, UL, and total leukemias, respectively. The AAIRs in El Salvador could not be calculated because the fourth age stratum in El Salvador included children only from 0–11 years old. The incidence rates for the Salvadoran group of 0–11 year olds were 34.2, 7.1, 0.6, 0.2, and 43.2 per million children for ALL, AML, CML, UL, and total leukemias, respectively.

**Conclusion:**

Reported AIRs for each age group in El Salvador were similar to those from other American countries. The AAIR of ALL in Mexico City is one of the highest reported for North America.

## Background

Leukemias are the most frequent type of cancer in childhood [1], the incidence of which varies depending on the area of the world where they are studied [2]. An elevated frequency of acute leukemias has been reported in populations of Hispanic origin [3-5]. For example, in Costa Rica, the highest incidence rate in the world for acute leukemias has been reported: 56 per million of children under the age of 15 years [5]. In California and Texas, two states of the U.S. which have a Hispanic component predominately of Mexican origin [3], the incidence rates for ALL and for AL in general have been found to be greater than those previously reported for Mexico City [6-8]. However, because the studies carried out in Mexico City have been retrospective, it is possible that a under-register of the infirmity may have existed in Mexico City. In Florida, the state with the highest predominance of Hispanics of Caribbean and Central American origin, the incidence rate of ALL was higher than that reported for the Caucasian population and similarly elevated to that reported for Costa Rica. For the Central American country of El Salvador, there is no prior information on the incidence of acute leukemias.

In developing countries, there are few data on the frequency of leukemias during childhood [5,9] because these countries have not had the infrastructure required to keep reliable records. Some international studies have produced information from some developing countries, particularly in Latin America [10]; however, this information has certain constraints with respect to its validity and coverage in representing the populations [11-15] Of the studies carried out in Mexico City, an important increase in the incidence of ALL, but not of AML, has been reported. In 1991, an incidence of 22.2 per million of children under 15 years of age was reported [6]. From the data from the Instituto Mexicano del Seguro Social, the Medical Center having the greatest coverage of the population in the whole of the country, an incidence rate was reported of 29.1 per million of children under 15 years of age who were residents of Mexico City and who had the right to receive attention from this Institution [7]. Given the retrospective nature of these studies, it is possible that there was underestimation of the incidence rate of ALL. On the other hand, when taken into account that, in the different states in the U.S. which have a high component of Hispanics of Mexican origin, the incidence rate of acute leukemias is the highest in the world, one may consider it important to perform a prospective population-based study in Mexico City that would determine if Mexican children, residing in this City, had an incidence rate similar to those reported for Texas and California [3].

Prospective records of those children treated for leukemia was initiated in El Salvador in 1994 and in Mexico City in 1996 [16,17]. In Mexico City, the record was kept in the hospitals of the Instituto Mexicano del Seguro Social (IMSS), an institution that attends about 50% of the population inhabiting Mexico City [7]. Because the IMSS keeps a register of the population that has a right to receive medical attention at its facilities, the base population is known and it is feasible to obtain the incidence rate for the population under 15 years of age. In El Salvador, because the record was kept by the Hospital Nacional de Niños "Benjamín Bloom" (BB), the only center in the country which attends all children, younger than 12 years with oncologic disease, data on the population base which was necessary for calculating the incidence rate was available. Treatment of children with leukemia is completely free of charge both in El Salvador and at the IMSS in Mexico City, thus allowing a greater coverage for children with this disease [16,17].

This work presents the incidence of leukemia in children younger than 12 years, because this is the age range admitted in the BB and data for children younger than 15 is used for Mexico City. In this report, for purposes of analysis, the data from 1996–2000 were used, with 1996 being the first year of record keeping in Mexico City and 2000 being the most recent year for data reported from El Salvador.

## Methods

### Design

Population-based data.

### Hospitals

The Hospital Nacional de Niños "Benjamín Bloom" (BB), located in San Salvador, capital of El Salvador; is a tertiary care center and is the only hospital in that country that has a pediatric hematology-oncology service with pediatric hematology staff. Being the only domestic reference center for pediatric oncologic diseases, it is the only hospital to which children with presumed leukemia diagnosis are referred for treatment. For this reason and because treatment is completely free of charge [16], no case of presumed childhood leukemia should be missing from the records. Children from other countries (Nicaragua, Honduras, etc) were excluded from the numerator.

The hospitals included in the Mexico City study were the Pediatric Hospital of the Centro Médico Nacional "Siglo XXI" (HP) and the General Hospital of the Centro Médico Nacional "La Raza" (HG). Children who were attended in the pediatric hematology service and who resided in Mexico City were included in both hospitals. There are accurate records on the population that has a right to this service because the population attended by this institution in Mexico City is formed by workers whose personal data are registered. Only those children who were Mexican nationals and whose parents were residents of Mexico City were included in the numerator.

### Diagnosis

Once diagnosed with presumed leukemia, a child was referred to one of the hospitals where trained staff did a comprehensive follow-up of the case to either discard or confirm the diagnosis of leukemia. In the BB, infirmary staff trained for that activity carried out this work. In all cases, the diagnosis was confirmed with bone marrow smear, and histochemical tests (myeloperoxidase, sudan black B reaction, esterases, periodic acid Schiff (PAS) reaction, and acid phosphatase) were performed to differentiate the types of leukemia. In the cases in El Salvador, the staff of St. Jude Hospital confirmed some diagnostic tests [16].

The types of leukemia were divided according to the five-group morphological classification of the international classification of childhood cancer. Only four of the types were found in this study: a) Acute lymphoid leukemia (ALL) (9820–9827, 9850); b) acute myeloid leukemia (AML), the preferred terminology instead of "acute nonlymphocytic leukemia" [18] (9840, 9841, 9861, 9864, 9866, 9867, 9891, 9894, 9910); c) chronic myeloid leukemia (CML) (9863, 9868); and e) unspecified leukemias (UL) (9800–9804) (International Classification of Disease for Oncology, ICD-O2; 1990) [19]. The codes were reviewed with the Child-Check program [19] to corroborate that there were no duplicate records or inconsistent data. A database was generated to record age, sex, residency, year of diagnosis, and clinical manifestation of the patients.

### Populations

Because, in El Salvador, only children under the age of 12 are attended at the BB, our analysis was limited to that cohort, instead of that conventionally used in a study of this type, i.e., children younger than 15 years [2,10]. The denominators to calculate the rates were obtained by every year from the General Direction of Statistics and Census (Dirección General de Estadística y Censos), which is the only government source that calculates the estimates of population in El Salvador. The population younger than 12 was 1,756,513; 1,764,422; 1,787,064; 1,808,946, and 1,829,146 for the years 1996 to 2000, respectively. The last available census in El Salvador dates from 2000.

In Mexico City, the denominators were obtained from the Coordination of Planning and Medical Information of the IMSS, which is the only government source that calculates all the populations with access to medical attention provided by the IMSS [20]. This information is updated every year by the IMSS. The recorded population younger than 15 years in Mexico City was 786,754; 832,199; 765,268; 806,043; and 881,887 for the years 1996 to 2000, respectively. The recorded population of children in Mexico City appeared to fluctuate from year to year because only that population of workers who are under contract with a company are entitled to the care provided by the IMSS. Thus, the denominator reflects the change in the number of jobs created or lost each year.

### Analysis

Annual incidence rates (AIR) were calculated by age group, sex, and each type of leukemia. Also, the average annual incidence rate (AAIR) was calculated by sex and total for the whole study period. These rates were standardized by age through the direct method with the world standard population [21], with the AIR and AAIR reported per million of children under 15 years old. The AAIRs were calculated by using the total of cases found in the study period as numerator and the sum of the populations found in each year of the study as denominator.

Cases were stratified by age and assigned to one of four age strata: 1) <1 year; 2) 1–4 years; 3) 5–9 years, or 4) 10–14 or 10–11 years, for Mexico City and El Salvador, respectively.

Therefore, for El Salvador, it was not possible to calculate the AAIR accurately for the 0–14 year-old age group. In the study, an average rate over the whole period and for the 0–11 year-old age group was calculated. These rates were standardized by age by direct method and also with the world standard population.

## Results

During the study period, there were 375 children diagnosed with leukemia in El Salvador and 238 in Mexico City. Of total leukemias in El Salvador and in Mexico City, ALL represented 80.5% and 76.9%; AML, 16.8% and 18.1%; CML, 1.6% and 4.2%; and UL, 1.1 and 0.8%, respectively.

The standardized rate by age of all leukemias in both countries was higher in boys than in girls, with a more marked male:female ratio in El Salvador than in Mexico City: 1.18 (46.8 per million children:39.5 per million children) vs. 1.01 (58.8 per million children:58.1 per million children). Whereas standardized rates by age in El Salvador revealed that all types of leukemias were more frequent in boys, this was not observed in AML or CML in Mexico City, where the frequency of both leukemias was higher for girls than that reported for boys (Tables I and II).

The low incidence of ALL in youngsters less than 1 year old found in El Salvador is notable, as is the fact that no case of AML in this age group was found in Mexico City.

The data for Mexico City, as those for El Salvador, showed that ALL had a peak between the ages of 1–4 years (Table 1 and 2). It is important to emphasize that, for the children in Mexico City, the age group with the highest AIR after that of the 1–4 year old group was the group of less than one year of age.

Among the subtypes of AML, the most frequent in Mexico City was AML M3 (01B9866) with nine cases, giving a frequency of 20.9% and an AAIR of 2.21 per million children. It is noteworthy that all these AML M3 cases were females. Unfortunately, in El Salvador, the frequency of AML M3 could not be assessed, as the major part of AML was classified only in the general category 01B9861.

## Discussion

Because there are only two important reviews that summarize the high incidence rates of leukemias in children reported in the world up to 1999 [1,10], it has been very difficult to obtain reliable data on the incidence of cancer in children, including leukemias, in developing countries. In fact, the review of Parkin *et al*., carried out in 1999, did not include data from either El Salvador or Mexico City because there were no reliable data from these areas at the time (1980 to 1989) at which they performed their study [10].

There are some issues of the present study that must be emphasized. In this study, statistics were not performed to compare the rates in Mexico City with the rates in El Salvador over time, because this was not the aim. The inclusion of the aforementioned institutions helped guarantee that all children diagnosed with leukemia during the studied period were included. This is due to the fact that, because BB is the only hospital in El Salvador that attends children with leukemia, any child with presumed leukemia is referred to that institution [16]. Similarly, in Mexico City, HP and HG are the only hospitals of the IMSS that attend the children with leukemia whose parents have rights to receive medical services from IMSS [17].

Even if there had been an under-registration of cases because some could not be diagnosed (e.g., patients dying before leukemia diagnosis is confirmed), the incidence rates in children of 0–11 years old found in El Salvador are similar to those reported in North America, which range from 36 to 45 , and with rates from South and Central America, which range from 29.0 to 57.9 per million children [10].

In Mexico City, there are various institutions outside the IMSS system that can attend children with cancer, particularly leukemia. However, such private institutions represent an economic burden for the family of the patient, in that the treatment for patients with leukemia lasts several years, with the possibility of expenses totaling almost $65,000 USD [22]. Therefore, it is less probable that the family of a child with leukemia would turn down access to the free service offered by HP and HG, opting instead for a costly private institution. For this reason, we think that cases in Mexico would not be underestimated due to patients being treated outside the IMSS system.

Mexico is a developing country with a high rate of child mortality in general. The absence of AML cases in infants of less than 1 year in Mexico City is notable because this is the peak age for AML in most developed countries [1,23], and suggests the probability that children younger than 1 year with AML die before being diagnosed. In contrast to ALL, AML seems to have no worse prognosis in children younger than 1 year [24,25], indicating that all children with AML, regardless of age, run the same risk of not being diagnosed. This increases the possibility of an apparently different pattern of age of AML onset in Mexico City. Little can be argued about the delay of diagnosis in these diseases, due to fast evolution of the disease and short-term mortality when no prompt management is provided; thus, acute leukemias are considered as the reference point for time of diagnosis [26].

As to the validity of measurements, recommended international morphological criteria were used in this study, and all measurements were performed by staff highly specialized in diagnosis and management of children with leukemia [7,16].

This study did not aim to assess incidence trend over time, a parameter that is affected by change(s) in diagnostic criteria [27], which were not a variable in this study. Since the period under study was too short to assess the incidence trend of leukemia, no analysis was done in this respect. However, this study evidences once again that choosing only one year to report a disease incidence may result in an over- or underestimation of the incidence of that disease [2]. For example, the observed incidence rate in Mexico City for the year 1996 in this study was much higher than those in the following years, suggesting that this might have been a completely random condition.

Although the standardized rates reported in this study were higher than those previously reported for Mexico City, the prior studies [6-8,26,28] were retrospective and therefore are not comparable to the present study. However, the possibility that the incidence of ALL is increasing, particularly in Mexico City as reported in these studies, must be considered [6,8]. This condition has been observed in various areas of Mexico City [8] and is associated with the more polluted areas and with farming areas [6,8].

The elevated incidence of ALL in children of Mexico City agrees with those found in other reports that show that ALL are more frequent in populations of Hispanics [3-5]. Table 3 shows that the AAIR of ALL is the highest reported for the population of Hispanic origin, with the value for the Hispanic populations of Texas being the highest, followed by that for Florida. Note that the majority of Hispanics in Texas are of Mexican origin[4]. The AAIR for ALL reported by SEER for Hispanics for the period 1992–1998 is very similar to that reported for Mexico City and agrees with the fact that, of the Hispanic children included in this report of SEER, 66% come from the Los Angeles register [29], where the great majority of the Hispanic population are of Mexican origin [4].

It is noteworthy that the AAIR of the age peak of ALL in boys in Mexico City is very high (82.4 per million in boys), an is higher than that reported for Costa Rica (76 per million in boys) and by SEER (76.7 per million in both sexs) [5,30]. However, the AAIR reported for Hispanics in general in Florida for the 0–4 year-old age group is 87.6 per million, which to our knowledge is the highest AAIR reported in the literature [4]. This finding is important because this peak in age is related with the higher frequency of ALL with the B-cell precursor immunophenotype. Although such data were not reported in this study, this immunophenotype is thought to be more frequent in developed countries [1] and, in addition, is related with genetic rearrangements such as TEL/AML1 that occur in the child during the intrauterine stage [31].

On the other hand, this age peak had been observed in relation to high socioeconomic status and population mixing [1]. The mix of urban and rural populations has been associated with the development of ALL [32,33] and especially with the cases that occurred in children under the age of 5 years [1]. Those states in the U.S.A. that have reported an elevated AAIR for ALL have an elevated level of immigration. The greater portion of Hispanics that emigrate to these zones come from rural regions. Mexico City has a similar situation, in that it constantly receives an influx of people from rural zones. Although, in this study, patients were determined to be residents of Mexico City, neither the place from which their parents came nor the length of time that the children resided in Mexico City was investigated. It will be important to investigate in the future if the mix between urban and rural populations is related with the very high incidence of ALL in Mexico City and in Hispanics in the U.S.A.

Another factor to be considered is the role of pesticides, whether occupational exposure of parents or exposure of children in the home. Also, we must not lose sight of the activities that Hispanic immigrants in the U.S.A. may perform, such as those related to the cultivation of crops, especially in areas of Texas where a higher frequency of ALL has been reported [34]. In Mexico City, the major zones with highest incidence rate of ALL are the zones in the South of the City where a portion of the population still carries out agricultural activities [8]. In a study carried out in Mexico City, an important relation was found between exposure to pesticides and the risk of developing AL [35]. In an ecological study realized in California, in which 36% of the cases with cancer were Hispanic children, it was found that the use of "Propargite", a pesticide used primarily in orchards and vineyards to control mites, was related to the higher rates of childhood leukemia [36].

The standardized incidence rate of ALL for children under 12 years of age in El Salvador was found to be less than the AAIR reported for Costa Rica or for Florida, where the highest percentage of Hispanics are of Caribbean and Central American origin, but this rate is relatively higher than that reported by SEER for all races [30]. This comparison has to be taken with some reservation because the AAIRs of SEER are reported for children under 15 years of age, whereas that of El Salvador is for only those under 12 years of age.

The frequency of AML M3 in the Hispanic population has been reported to be higher than those for other populations [37]. This has been observed both for children and for adults [3,37-39] (Table 4). In the present study, it was found that, in Mexico City, the frequency was 21%, a value consistent with the data that the frequency of AML M3 is higher in a population of Hispanic origin. The present study has the advantage over other studies that were carried out in Latin America because it is population-based. One study has proposed that the elevated frequency of AML in the Hispanic population may be related not only genetic factors but also nutritional ones [38].

It is important to emphasize that, despite recent data published on the incidence of leukemias in Latin America [5,40] or in Hispanics from the USA [29], those study periods were prior to those analyzed here. Finally, it can be stated that the rates here presented are similar to those reported for Latin America, thereby supporting the reliability of these results. This is the start of a project that intends to join efforts in Mexico City, El Salvador, Honduras, Guatemala, Costa Rica, and Nicaragua to keep records of cancer in children, which will be highly reliable and which, therefore, can provide valid and timely data on the great concern that child cancer represents in the Latin American countries.

## Conclusion

The AAIRs reported for Mexico City and the rate in children from 0–11 years old in El Salvador were similar to those that have been reported by other Latin American countries. The AAIR of ALL in Mexico City is one of the highest in the world.

## Competing interests

The author(s) declare that they have no competing interests.

## Authors' contributions

JMMA conceived and designed the study, analyzed the data and wrote the first draft of the manuscript. MB and AFG designed the study, analyzed the data, and provided guidance to all aspects of this project. RL, SJO, GR, MLPS, GGM, RBR, AOF, MOA and MCMG registered, recorded, and analyzed the data. All authors read and approved the final manuscript.

## Pre-publication history

The pre-publication history for this paper can be accessed here:



## Figures and Tables

**Table 1:** The incidence of leukemia in children aged 0–14 years in Mexico City from 1996 to 2000.

**1996 to 2000**									**Number of cases by year**				
		
	**Sex**	**n**	**AAIR**	**< 1 year**	**1–4 years**	**5–9 years**	**10–14 years**		**1996**	**1997**	**1998**	**1999**	**2000**
		
**ALL**	M	103	49.6*	60.2	82.4	49.9	19.5	*n*	49	33	33	42	26
	F	80	40.1*	36.7	45.9	41.8	33.9	*AAIR*	62.3	39.6	43.1	52.1	29.5
	T	183	44.9*	48.8	64.6	45.9	26.6						
		
**AML**	M	13	6.3*	0	10.5	6.9	3.0	*n*	9	4	11	7	12
	F	30	15.0*	0	16.5	14.4	17.0	*AAIR*	11.4	4.8	14.4	8.7	13.6
	T	43	10.6*	0	13.4	10.6	9.9						
		
**CML**	M	5	2.4*	8.6	0	1.4	4.5	*n*	2	3	0	4	1
	F	5	2.5*	0	5.5	1.4	1.5	*AAIR*	2.5	3.6	0	5.0	1.1
	T	10	2.5*	4.4	2.7	1.4	3.0						
		
**UL**	M	1	0.5*	0	0	1.4	0	*n*	1	0	0	1	0
	F	1	0.5*	0	0	0	1.5	*AAIR*	1.3	0	0	1.2	0
	T	2	0.5*	0	0	0.7	0.8						

AAIR: Average Annual Incidence Rate

M = male; F = female; T = total; AAIR = average annual incidence rate per million. AML = Acute myeloid leukemia; ALL = Acute Lymphoid leukemia; CML: Chronic Myeloid Leukemia; UL = Unspecified leukemias.* Age standardized rate per million children.

**Table 2:** The incidence of the leukemias in children younger than 12 years in El Salvador from 1996 to 2000.

**1996 to 2000**									**Number of cases by year**				
		
	**Sex**	**n**	**Rate over the entire period**	**< 1 year**	**1–4 years**	**5–9 years**	**10–11 years**		**1996**	**1997**	**1998**	**1999**	**2000**
		
**ALL**	M	162	36.1*	4.9	49.6	36.3	20.1	*n*	54	64	61	60	63
	F	140	32.3*	15.1	47.3	23.0	29.7	*Rate over the entire period*	30.7	36.3	34.1	33.2	34.4
	T	302	34.2*	9.9	48.4	29.7	24.8						
		
**AML**	M	37	8.1*	0	9.4	9.2	7.2	*n*	19	12	12	9	11
	F	26	6.0*	7.6	9.1	3.4	4.4	*Rate over the entire period*	10.8	6.8	6.7	5.0	6.0
	T	63	7.1*	3.7	9.2	6.3	5.8						
		
**CML**	M	3	0.6*	0	0	1.1	1.4	*n*	0	0	1	3	2
	F	3	0.6*	0	0	1.1	1.5	*Rate over the entire period*	0	0	0.6	1.7	1.1
	T	6	0.6*	0	0	1.1	1.5						
		
**UL**	M	3	0.7*	2.4	1.2	0	0	*n*	0	0	1	1	2
	F	1	0.2*	2.5	0	0	0	*Rate over the entire period*	0	0	0.6	0.5	1.1
	T	4	0.5*	2.5	0.6	0	0						

AAIR: Average Annual Incidence Rate. M = male; F = female; T = total. AML = Acute myeloid leukemia; ALL = Acute Lymphoid leukemia; CML: Chronic Myeloid Leukemia UL = Unspecified leukemias.

* Age standardized rate per million children.

**Table 3:** Comparison of annual age-adjusted rates of lymphoid leukemias per million for Mexican and Salvadoran children with those for Hispanic children from three U.S. Cancer Registries and for children from Costa Rica

***Leukemia type***	***SEER 2001 All races ***(30)	***Mexico City, IMSS***	***Texas ***(34)	***California ***(3)	***SEER 1992–1998 Hispanics ***(29)	***El Salvador***	***Florida ***(4)	***Costa Rica ***(5)
Lymphoid leukemia	33.2	44.9	52.0	44.0	43.0	34.2*	49.7	43.1

IMSS = Instituto Mexicano del Seguro Social.

* This rate is only for children 0–11 years old.

(References)

**Table 4:** Comparison of the proportion of promyelocytic leukemias for Mexican children with Hispanic children from other countries and cities.

	Adults				Children			
***Leukemia type***	***LAC-USC ***(37)	***LA County and AML study ***(37)	***Peru ***(38)	***Puebla, Mexico ***(39)	California* (3)	***Puebla, Mexico ***(39)	***Mexico City, IMSS***	Range in non-Hispanic populations** (37)

Promyelocytic leukemia	37.5%	24.3%	22.0%	20.0%	8.3%	30.0%	21.0%	3–13%

LAC-USC = Los Angeles County-University of Southern California; LA: Los Angeles; AML = acute myeloid leukemia; IMSS = Instituto Mexicano del Seguro Social.

* This is a population-based study in which a frequency of 3.8%was found for non-Hispanic children.

** This range is based on proportions found in series of cases.

(References)
